# Characteristics of first-time varenicline users – A cross-sectional study in Finnish quitters

**DOI:** 10.1186/s12889-017-4248-1

**Published:** 2017-04-19

**Authors:** Hanna E. Tervonen, Juha H. O. Turunen, Christine L. Baker, Juha Laine, Kari Linden

**Affiliations:** 1 School of Health Sciences, Centre for Population Health Research, University of South Australia, GPO Box 2471, Adelaide, SA 5001 Australia; 2 Research Department, Farenta Oy, Äyritie 16, FI-01510 Vantaa, Finland; 3 Pfizer Inc, E. 42nd Street, New York, NY 10017 USA; 4 Pfizer Oy, Tietokuja 4, FI-00330 Helsinki, Finland

**Keywords:** Smoking, Smoking cessation, Varenicline, Tobacco use disorder, Finland

## Abstract

**Background:**

Varenicline is an efficacious medicine for smoking cessation (SC) but little is known about the characteristics of varenicline users. This study examined the characteristics of first-time (naïve) varenicline users in Finland and compared those who had previously used SC pharmacotherapy to those who were trying SC pharmacotherapy for the first time.

**Methods:**

A cross-sectional survey was conducted in Finnish community pharmacies between February 2014 and January 2015. Pharmacy customers purchasing a varenicline starter package for the first time ever were asked to complete a questionnaire or to participate in a structured interview conducted by the pharmacist (identical questions). The questionnaire included questions about demographic characteristics, smoking habits, previous cessation attempts and factors associated with varenicline use.

**Results:**

Altogether 98 people completed the survey. The majority were daily smokers (96%, *n* = 94), with a history of over 10 years of regular smoking (94%, *n* = 92), and a strong/very strong nicotine dependence (67%, *n* = 66). Half of the participants (54%, *n* = 53) were trying a SC pharmacotherapy for the first time. Demographic characteristics and smoking habits were similar between first-time and previous users of SC medications (*p* > 0.05). Health centers (42%, *n* = 41) and occupational health care clinics (37%, *n* = 36) were the most common sources of varenicline prescriptions. The majority of participants received the prescription for varenicline after mentioning their desire for quitting to a physician (70%, *n* = 69).

**Conclusions:**

Considering the relatively large proportion of SC naïve medicine users among new users of varenicline, smokers who have previously been reluctant to quit smoking, to use other pharmacological SC interventions, or perhaps unaware of these options may be interested in attempting cessation with varenicline. Most participants made the initiative to discuss their smoking with the physician, which led to varenicline prescribing. This suggests that physicians may not satisfactorily recognize their patients’ nicotine dependence and desire to quit, and they should more actively support patients’ smoking cessation.

## Background

Smoking is a leading cause of premature and preventable mortality worldwide [1]. In the European Union, the prevalence of current smoking was 26% in 2014 [2]. In Finland, 17% of males and 14% of females were daily smokers in 2014 [3]. During the last 12 months, 35% of males and 42% of females had attempted to give up smoking. There are several effective pharmacological treatment options which can help people to quit smoking [4]. These options include nicotine replacement therapy (NRT), bupropion and varenicline which have all been shown to improve the chance of quitting. The combination of pharmacotherapy and behavioral support increases cessation success in comparison to usual care or minimal intervention such as brief advice [5]. Pharmacotherapy has been shown to be a cost saving option for smoking cessation [6]. Physicians have an important role in supporting smoking cessation because they can prescribe the most effective smoking cessation medicines [4]. Even a short intervention by a physician is shown to be effective [7–10]. According to the Finnish Current Care guideline on smoking cessation, all smoking patients should be offered the most effective smoking cessation therapy that is appropriate for them [11]. Factors associated with the use of SC pharmacotherapy include heavy smoking, higher income, older age, being female and participating in an education program about smoking prevention or cessation [12, 13].

Despite the existence of efficient treatment options, many smokers attempt to quit without any pharmacotherapy [12–14]. This may be due to concerns over safety, lack of knowledge on the most efficient quitting aids, medicine costs, reimbursement policies, or low smoking cessation activity by physicians and other health care professionals [9, 15, 16]. Only 3–5% of self-quitters achieve prolonged abstinence for 6–12 months after a cessation attempt [17]. Smokers may have several unassisted quit attempts before being ultimately successful; although the proportion of unassisted attempts has decreased over time as a variety of SC products have become available [18]. Nevertheless, there is evidence that buying over-the-counter NRT without any behavioral support is associated with a similar likelihood of successful cessation than not using any cessation aids [19]. Therefore, physicians and health care personnel should actively provide information and support to smokers attempting to quit smoking [8, 9, 20].

Varenicline is a selective nicotinic receptor partial agonist which is licensed for smoking cessation and can be purchased only with a prescription [11, 21]. According to the United States (US) Public Health Service Clinical Practice Guideline, varenicline is appropriate as a first-line medication for smoking cessation [8]. In Finland, varenicline has been on market since 2006. However, the use of pharmacotherapy for smoking cessation is relatively uncommon in Finland; only 2% of adult smokers attempting to quit smoking had used a cessation medication (bupropion/varenicline) prescribed by a physician during the previous year [22]. In Finland, varenicline was granted with a basic reimbursement (35% of the medicine price) in June 2014 for motivated people with strong nicotine dependence who have previously failed to quit with other support actions. In general, little is known about the characteristics of the users of varenicline [23–27].

The aim of this study was to examine the characteristics of first-time varenicline users in Finnish community pharmacies and to compare the characteristics of those who had previously used SC pharmacotherapy to those who were trying SC pharmacotherapy for the first time.

## Methods

A cross-sectional survey was conducted in 46 community pharmacies located in 37 towns across Finland (representing about 8% of 600 Finnish pharmacies). The selection of pharmacies was based on a convenience sample. Approximately 80 pharmacy owners, whose contact information was readily available, were initially asked to participate in the study. The selected pharmacies were distributed across Finland, with multiple pharmacies included only from bigger towns in order to ensure geographical coverage. Data were collected between February 2014 and January 2015. To be included in this study, the pharmacy customer had to be at least 18 years old and purchasing a varenicline starter package for the first time ever. Eligible participants were asked to fill in a self-administered electronic questionnaire with a tablet computer or alternatively complete the electronic questionnaire as a structured interview conducted by the pharmacist. The questionnaire included questions about demographic characteristics (sex, age), smoking habits (smoking history, 2-question Fagerstrom Test/Heaviness of Smoking Index) [28], previous cessation attempts (number of attempts, previously used cessation aids) and factors associated with the use of varenicline (source of prescription, factors associated with the decision to quit and to use varenicline). Score of ≥3 from the 2-question Fagerstrom Test/Heaviness of Smoking Index was regarded as strong or very strong nicotine dependence [11].

The survey was conducted in accordance with recommendations made by the Finnish National Advisory Board on Research Ethics. According to Finnish guidelines, ethics committee approval is not required for surveys to adults [29]. All potential participants were provided with detailed written information about the survey. Completing the questionnaire or answering the interview was considered as consent to participate. Any identifying information of the respondents was not asked or recorded.

The results are presented as means, standard deviations (SD) and frequency distributions for the overall group of varenicline-naïve users as well as the subgroups of those using SC pharmacotherapy for the first time versus previous users of SC pharmacotherapy (nicotine replacement therapy and/or prescription medicines but not varenicline). The Fisher’s Exact test was used to examine differences in variable distributions between these subgroups. Student’s t-test was used to compare mean age between these groups. The data were analyzed using IBM SPSS Statistics Version 21.0 (IBM Corp. Armonk, NY, US).

## Results

Altogether 98 people participated in the study and completed the survey. Of these, 56% (*n* = 55) were males and the mean age of participants was 49 years (range 25–81 years) (Table 1). Almost all participants were daily smokers (96%, *n* = 94) and had a history of over 10 years of regular smoking (94%, *n* = 92). According to the 2-question Fagerstrom Test/Heaviness of Smoking Index, 67% of participants (*n* = 66) had a strong or very strong nicotine dependence. Almost half of participants smoked 11–20 cigarettes per day (46%, *n* = 45) and 24% of participants smoked their first cigarette within 1–5 min after waking up (*n* = 23). The majority of participants had tried quitting 1–4 times (including the current attempt) (82%, *n* = 79). For 20 participants, the current quit attempt was the first attempt ever (21%).

**Table 1:** Characteristics of the study sample and comparison between first-time and previous users of smoking cessation pharmacotherapy

	All (*n* = 98)	First-time users of smoking cessation pharmacotherapy (*n* = 53)	Previous users of smoking cessation pharmacotherapy (*n* = 45)	*P* value^a^
Sex, *n* (%)^b^				0.686
Males	55 (56.7)	29 (54.7)	26 (59.1)	
Females	42 (43.3)	24 (45.3)	18 (40.9)	
Mean age (SD)	49.4 (13.5)	48.1 (13.7)	50.9 (13.3)	0.615
Proportion of daily smokers, *n* (%)	94 (95.9)	52 (98.1)	42 (93.3)	0.331
Years of smoking, *n* (%)				1.000
0–10 years	6 (6.1)	3 (5.7)	3 (6.7)	
Over 10 years	92 (93.9)	50 (94.3)	42 (93.3)	
Proportion of participants with strong/very strong nicotine dependence, *n* (%)^c^	66 (67.3)	34 (64.2)	32 (71.1)	0.521
Number of cigarettes smoked daily, *n* (%)				0.364
1–10	22 (22.4)	13 (24.5)	9 (20.0)	
11–20	45 (45.9)	20 (37.7)	25 (55.6)	
21–30	22 (22.4)	14 (26.4)	8 (17.8)	
More than 30	9 (9.2)	6 (11.3)	3 (6.7)	
Time between waking up and smoking the first cigarette, n (%)				1.000
1–5 min	23 (23.5)	12 (22.6)	11 (24.4)	
More than 5 min	75 (76.5)	41 (77.4)	34 (75.6)	
Number of overall quit attempts, *n* (%)^d^				0.030
1–4	79 (82.3)	48 (90.6)	31 (72.1)	
5 or more	17 (17.7)	5 (9.4)	12 (27.9)	

^a^Fisher’s Exact test and Student’s T-test were used to compare the first-time and previous users of smoking cessation pharmacotherapy

^b^Data missing for one person

^c^Measured using the 2-question Fagerstrom Test (Heaviness of Smoking Index): score of 3 or more out of 6 means strong or very strong dependence

^d^Including the current attempt. Data missing for two people

There were 53 participants (54%) who were using SC pharmacotherapy for the first time. The demographic characteristics, smoking history and current smoking habits were similar between the groups of first-time and previous medication users (all *p* values >0.05) (Table 1). Among first-time medication users, 38% (*n* = 20) smoked more than 20 cigarettes per day compared with 24% (*n* = 11) among previous medication users but the difference was not statistically significant (*p* = 0.349). The only statistically significant difference was the number of previous quit attempts; a larger proportion of participants who had previously tried SC pharmacotherapy had 5 or more quit attempts (28%, *n* = 12) compared with participants who were using SC pharmacotherapy for the first time (9%, *n* = 5) (*p* = 0.030).

Among the 45 participants (46%) who had previously tried SC pharmacotherapy, the most commonly used interventions were nicotine gum (76%, *n* = 34), nicotine patch (47%, *n* = 21) and nicotine lozenges (44%, *n* = 20). According to participants, the most important reason for previous use of NRT products was the convenient prescription-free accessibility (48% of 42 participants who had previously used only NRT). One fifth of the previous NRT users had not heard about prescription medicines for smoking cessation before their current attempt to quit (21%).

The majority of participants received the prescription either in a health center (42%, *n* = 41) or occupational health care clinic (37%, *n* = 36). The majority of participants received the prescription for varenicline after mentioning their desire for quitting to a physician (70%, *n* = 69). In 30% of cases (*n* = 29), the physician made the suggestion for quitting with varenicline. The most frequently mentioned factor contributing to the decision to quit smoking was concern about personal health (93%, *n* = 91) followed by encouragement from family or friends (32%, *n* = 31) and encouragement from physician (31%, *n* = 30).

## Discussion

This study provides new information about the characteristics of varenicline users in Finland, where the reimbursement status of varenicline was recently granted. The majority of the participants were daily smokers who had been smoking for more than 10 years and had strong nicotine dependence. Interestingly, more than half of the participants were trying a SC pharmacotherapy for the first time. A study from the United Kingdom examined the characteristics of varenicline users and reported a markedly higher proportion of varenicline users (87%) who had previous experience with NRT products [23]. Also in our study, NRT products were the most commonly used pharmacological cessation aid for those having previous SC medication experience. Health centers and occupational health care play an important role in smoking cessation and approximately 80% of the varenicline prescriptions were prescribed in these locations.

The relatively large proportion of first time users of SC pharmacotherapy in this group of varenicline-naïve users may imply that people who have previously been reluctant to quit smoking or use pharmacological interventions, or who lacked awareness of these smoking cessation aids may be interested in attempting smoking cessation with varenicline. Some quitters may have distrust in NRT’s efficacy or safety profile [15, 16]. Prescription medicines, such as varenicline, can be advertised only to physicians in Finland. The findings from this study on the characteristics of the sample (daily smokers, long smoking histories, strong/very strong nicotine dependence) are in line with previous studies which have shown that heavy smokers are more likely to use pharmacotherapy for smoking cessation [12, 13]. Studies examining specifically varenicline users have also reported that varenicline is more likely to be used by heavy smokers [27] and smokers with high nicotine dependence [30]. An Australian study examined the characteristics of people who filled a prescription for varenicline and reported a well-balanced gender mix (55% females), mean age of 48 years, long smoking histories, high proportion of daily smoking and a high number of cigarettes smoked daily (mean 22 cigarettes) [31]. These characteristics resemble the characteristics of our sample, with the exception of lower proportion of people with no previous quit attempts (8%) compared to 21% in our study.

Our results suggest that varenicline is more frequently used for more dependent quitters and, at least to some extent, in the second line of smoking cessation treatment which is in accordance with the Finnish reimbursement requirements and the previous literature [25, 30]. From the clinical viewpoint and on the basis of the Finnish and other smoking cessation guidelines, varenicline use is not limited to the heavily dependent quitters [8, 9, 11]. There is evidence that the level of baseline nicotine dependence had no effect of the efficacy of varenicline in comparison to placebo [32]. Demographic characteristics and smoking habits were similar in both groups, i.e., those using pharmacological intervention for the first time and those with previous pharmacological attempts which is in accordance with earlier findings in the US [30]. The only statistically significant difference between these groups was the number of overall quit attempts, with previous users of SC pharmacotherapy having more quit attempts. The lack of statistically significant differences between the groups may be partly explained by the small sample size.

Most participants received the prescription for varenicline after mentioning their desire for quitting to a physician. In contrast, the physician first made the recommendation to prescribe varenicline only in less than one third of patients. Other studies have reported similar findings. For example, in a study by Joshi et al. (2010) over half of the physicians reported that they discussed smoking cessation with their patients while only 15% of the patients agreed with this [10]. Further in that study, over three quarters of the physicians reported that they initiated the discussion while 63% the patients considered that they took the initiative. In the present study, only a third of the respondents felt that their quit attempt was encouraged by their physicians. This is in alignment with a recent national Finnish survey which reported that 39% of the people who had made a serious quit attempt had received advice from a physician [3]. Similarly, an Australian study reported that varenicline users did not receive an adequate level of support by physicians [31].

Encouraging smoking cessation is part of physicians’ responsibilities [8]. However, studies have shown that physicians rarely actively treat their patients’ nicotine dependence or support their smoking cessation [33]. The Finnish Current Care guideline on smoking cessation underlines physicians’ role in smoking cessation and the use of the most effective smoking cessation therapies for all quitters [11]. A recent Finnish study reported that physicians acknowledged the need to discuss about smoking with their patients and widely recognized nicotine dependence as a disease to be treated by a physician, but rarely provided practical cessation support; only 4% of the respondents prescribed a pharmacological cessation aid and 10% recommended NRT use [34, 35].

Some limitations need to be acknowledged. A small sample size complicates the use of statistical testing and may have caused our analyses to be underpowered. This also precluded the use of multivariable modelling. However, the primary aim of this study was to describe the characteristics of first-time varenicline users in Finland with less emphasis on statistical testing. Data were collected as part of normal clinical practice (real-world data) and we were not able to collect information on the number of all eligible smokers or the number of people who declined to participate in the study. Therefore, we cannot calculate a response rate or reliably estimate the representativeness of our sample. However, data were collected in 46 community pharmacies located in 37 towns across Finland providing a wide variety of different pharmacies and geographic locations. Given its exploratory aim, the study did not include a comparison group of smokers taking other SC treatments or smokers who tried quitting without medications. Therefore, we cannot compare the characteristics of varenicline-users to smokers attempting to quit without varenicline. In addition, our sample is unlikely to be representative of Finnish smokers in general or general clinical practice on smoking cessation in Finland because this study examined smokers who were taking varenicline and their recollections of their physicians’ practices. Further research on these topics is of interest.

## Conclusions

In Finland, varenicline appears to be prescribed as a first and second line smoking cessation treatment, typically for older working aged people with a strong nicotine dependence and long smoking history. Approximately half of the respondents had used other treatment options before varenicline, typically NRT for its easy availability. Over 70% of the varenicline-users took the initiative to discuss their smoking with their physician and only a third of the participants reported that their quit attempt was encouraged by their physician. This study shows that Finnish physicians could more actively ask about their patients’ smoking, diagnose nicotine dependence and initiate and support their patients’ smoking cessation attempts as guided by the national and international smoking cessation guidelines. This is critical because appropriate smoking cessation treatment is one of the most effective interventions in primary care and in several secondary care settings to enhance patients’ health and life expectancy [8–10].

## Abbreviations

NRT: Nicotine replacement therapy
SC: Smoking cessation
SD: Standard deviation
